# Impact of Tumor Geometry on Dose Distribution and Delivery Accuracy in Multi-Target Stereotactic Radiosurgery

**DOI:** 10.3390/brainsci16060571

**Published:** 2026-05-28

**Authors:** Hsiao-Mei Fu, Tsung-Yu Yen, Chia-Ting Lee, Ko-Hsin Hsiao, Yu-Po Shen, Shih-Ming Hsu

**Affiliations:** 1 Department of Biomedical Imaging and Radiological Sciences, National Yang Ming Chiao Tung University, Beitou District, Taipei 112, Taiwan; moos1028.b478@mmh.org.tw (H.-M.F.); yms851115@yahoo.com.tw (T.-Y.Y.); ctl.be14@nycu.edu.tw (C.-T.L.); 2 Department of Radiation Oncology, MacKay Memorial Hospital, Tamsui District, New Taipei City 251, Taiwanjojo19800819@yahoo.com.tw (Y.-P.S.); 3 School of Medicine, College of Medicine, MacKay Medical University, Sanzhi District, New Taipei City 252, Taiwan; 4 Hospice and Palliative Care Center, MacKay Memorial Hospital, Tamsui District, New Taipei City 251, Taiwan; 5 Department of Death Care Service, MacKay Junior College of Medicine, Nursing and Management, Sanzhi District, New Taipei City 252, Taiwan

**Keywords:** stereotactic radiosurgery, multiple brain metastasis, automatic lower dose objective, volumetric modulated arc therapy, quality assurance

## Abstract

**Highlights:**

**What are the main findings?**

- **Automatic Lower Dose Objective (ALDO) dual-impact:** ALDO improved dose conformity, whereas increased dose heterogeneity and hotspot formation were more pronounced in small-target configurations.
- **Verification variability:** In non-ALDO configurations, differences between portal dosimetry and film measurements were observed depending on target size, spatial position, and gamma criteria.

**What is the implication of the main findings?**

- **Clinical balancing**: Physicians and medical physicists must balance high-gradient benefits with internal hot spots in multi-metastasis planning when using the ALDO function in treatment planning.

**Abstract:**

**Objectives**: This study aimed to evaluate the influence of tumor geometry on dose distribution and delivery accuracy, and to assess the impact of the Automatic Lower Dose Objective (ALDO) function on dosimetric performance. **Methods**: Computed tomography images of a Rando anthropomorphic phantom were used to simulate intracranial multiple metastases. Two contour groups were generated on the same CT dataset, consisting of two spherical targets with diameters of 1 cm and 2 cm, respectively. For each group, target pairs were created with edge-to-edge separation distances ranging from 1 to 6 cm. Automated single-isocenter stereotactic radiosurgery plans were generated using the HyperArc workflow, both with and without the ALDO function. Dosimetric performance was evaluated using the RTOG conformity index, Paddick conformity index, gradient index, and homogeneity index. Patient-specific quality assurance was performed using electronic portal imaging device-based verification and radiochromic film dosimetry. Gamma analysis with multiple criteria was applied to assess the impact of target size and geometric separation on delivery accuracy. **Results**: The use of ALDO improved dose conformity and gradient performance but resulted in increased dose heterogeneity and higher hot spots. In non-ALDO configurations, the agreement between EPID portal dosimetry and film measurements varied according to target size, gamma criteria, and spatial position. For the 2 cm targets, EPID portal dosimetry generally demonstrated higher gamma passing rates than film measurements, whereas the 1 cm targets showed mixed results depending on measurement position and gamma criteria. These differences likely reflect the distinct detector characteristics and spatial sensitivities of the two QA methodologies. Larger discrepancies were observed under stricter gamma criteria and at off-axis positions, indicating potential influences of target geometry and high-dose gradient regions within the simplified phantom configurations evaluated in this study. **Conclusions**: Within the simplified two-target phantom configurations evaluated in this study, tumor geometric distribution significantly affects both dosimetric characteristics and QA outcomes in HyperArc SRS. Film measurements provide greater sensitivity, whereas EPID-PD alone may be insufficient for evaluating small-target high-gradient regions under strict gamma criteria.

## 1. Introduction

Stereotactic radiosurgery (SRS) has become an essential modality for the management of intracranial tumors, particularly in patients with brain metastases, where precise dose delivery is critical for preserving neurological function and quality of life [1,2]. In recent years, single-isocenter multiple-target (SIMT) techniques have enabled the simultaneous treatment of multiple lesions, improving treatment efficiency and reducing patient burden [3,4]. However, treating multiple targets within a single isocenter introduces additional planning and delivery complexity. In particular, the spatial relationship between tumors—including inter-target distance, relative positioning, and target size—may influence dose conformity, dose fall-off, and the extent of low-dose irradiation to normal brain tissue. Despite its clinical relevance, the specific influence of tumor geometry on dosimetric performance and delivery accuracy in simplified multi-target HyperArc configurations remains incompletely characterized.

Volumetric modulated arc therapy (VMAT)-based SIMT has become widely adopted for the treatment of multiple brain metastases. More recently, HyperArc (Varian Medical Systems, Palo Alto, CA, USA), an automated noncoplanar VMAT technique, has been introduced to enhance planning efficiency and consistency. By standardizing beam geometry, collimator settings, and collision avoidance strategies, HyperArc reduces inter-planner variability and streamlines the planning workflow [5,6]. Previous studies have demonstrated that HyperArc can achieve improved dose conformity, steeper dose gradients, and enhanced sparing of normal brain tissue compared with conventional VMAT approaches [7,8,9]. Similar advantages have also been reported in single-lesion SRS, where improved gradient characteristics and reduced low-dose spread were observed [10].

Despite these advantages, SIMT techniques remain sensitive to both geometric and mechanical factors. Targeting accuracy has been shown to decrease with increasing distance from the isocenter, although modern image-guided systems can maintain clinically acceptable accuracy within a limited range [11,12]. However, most prior investigations have primarily focused on mechanical accuracy rather than dosimetric consequences. Emerging evidence suggests that tumor spatial configuration may influence dose distribution. Closely spaced lesions may increase low-dose spread and reduce dose gradients because of overlapping dose distributions between adjacent targets, potentially resulting in dose-bridging effects [13,14]. Furthermore, Chen et al. reported that HyperArc improves dose conformity and reduces dose bridging, while also demonstrating correlations between tumor characteristics and dosimetric indices [9]. These findings indicate that tumor geometry may play a critical role in treatment planning for multi-target SRS.

Accurate verification of dose delivery is also essential in HyperArc-based SRS, particularly for small targets and complex multi-target geometries. Previous studies have shown that film-based dosimetry provides superior spatial resolution and greater sensitivity to dose variations compared to portal dosimetry, especially in high-gradient and off-axis regions [15]. However, the extent to which tumor geometric configuration influences discrepancies between different quality assurance (QA) modalities has not been fully explored.

From a clinical perspective, patient-specific QA remains a key component of safe SRS delivery. Portal dosimetry (PD) is widely used in routine practice due to its efficiency and convenience, but its ability to detect subtle geometric or off-axis dose discrepancies may be limited. In contrast, radiochromic films offer higher spatial resolution and may be more sensitive to small dose variations, particularly in regions with steep dose gradients. These differences raise important questions regarding the adequacy of current QA approaches for increasingly complex multi-target SRS plans.

In addition, the Automatic Lower Dose Objective (ALDO) function in HyperArc has been developed to facilitate consistent target coverage with reduced manual intervention [16]. While this feature improves planning efficiency, its interaction with tumor geometry and its potential influence on dose distribution and delivery accuracy remain unclear.

Therefore, this study aimed to investigate the influence of tumor size and spatial geometric distribution on dosimetric characteristics and delivery accuracy in single-isocenter SRS using the HyperArc system. By integrating dosimetric analysis with multi-modality QA evaluation, this work seeks to provide a more comprehensive understanding of geometry-related effects and to support the optimization of multi-target SRS in clinical practice.

## 2. Materials and Methods

### 2.1. CT Simulation and Target Contouring

Computed tomography (CT) images of a Rando anthropomorphic phantom were acquired to simulate intracranial multiple metastases. CT simulation was performed using a Philips Big Bore CT scanner (Philips Healthcare, Cleveland, OH, USA) with a slice thickness of 1 mm to ensure sufficient spatial resolution for stereotactic radiosurgery planning. The acquired CT dataset was imported into the treatment planning system for subsequent target contouring and plan generation.

To simulate multiple brain lesions, spherical target volumes were contoured within the brain region on the same CT dataset. Two contour groups were created, each consisting of two targets with diameters of 1 cm and 2 cm, respectively. The targets were positioned along a predefined axis, and the edge-to-edge separation distance between the targets was systematically varied from 1 to 6 cm to represent different spatial configurations.

### 2.2. Treatment Planning

Treatment planning was performed using the Eclipse treatment planning system (version 16.1, Varian Medical Systems, Palo Alto, CA, USA). Dose calculations were carried out using the Anisotropic Analytical Algorithm (AAA) with a calculation grid size of 1.25 mm. All plans were generated following the HyperArc workflow, which employs automated noncoplanar VMAT for SRS.

For all simulated cases, the prescription dose was set to 800 cGy per fraction for three fractions (800 cGy × 3 fx) representing a hypofractionated stereotactic treatment approach. For each target configuration, we created two plans, with and without the ALDO function, respectively. All plans were normalized to 100% without additional manual renormalization. The HyperArc planning approach utilizes a standardized arc arrangement, including one coplanar arc and three noncoplanar arcs, to achieve highly conformal dose distributions while minimizing dose to surrounding normal tissues. The noncoplanar arcs were delivered using couch angles of 45°, 315°, and 270°, according to the institutional HyperArc planning template.

All planning parameters were kept consistent between the two plan types to allow for a direct comparison of the dosimetric effects of the ALDO function. No additional maximum dose constraint was applied in the ALDO plans, in accordance with the default optimization strategy of the HyperArc system. The Automatic Lower Dose Objective (ALDO) function was designed to automatically achieve approximately 98% relative target coverage across all lesions during optimization [16]. In the non-ALDO plans, a relatively loose maximum dose constraint was manually applied to allow for optimization flexibility while maintaining clinically acceptable dose distributions. Both ALDO and non-ALDO plans utilized the SRS Normal Tissue Objective (SRS NTO) during optimization.

### 2.3. Dosimetric Evaluation

All treatment plans were delivered using a Varian TrueBeam Edge linear accelerator (Varian Medical Systems, Palo Alto, CA, USA) with 6 MV flattening filter-free (FFF) photon beams. The system is equipped with a high-definition 120-leaf multileaf collimator (HD120 MLC), consisting of 32 central leaf pairs with a width of 2.5 mm and 28 peripheral leaf pairs with a width of 5.0 mm at isocenter. Dosimetric performance was evaluated using standard indices commonly applied in stereotactic radiosurgery. The conformity of the treatment plans was assessed using the RTOG conformity index (CI) and the Paddick conformity index. Dose gradient characteristics were evaluated using the gradient index (GI), and dose homogeneity within the target was assessed using the homogeneity index (HI) based on the ICRU Report 83 definition [17,18,19].

All indices were calculated for each target across different geometric configurations and compared between plans generated with and without the ALDO function. These parameters were used to quantify the influence of target size and inter-target separation on dose distribution. For each configuration, dosimetric parameters were calculated separately for targets located on the left and right sides of the isocenter, and the average values were used for analysis.

All plans were generated under identical normalization conditions, and D95% target coverage was maintained at 100% for all configurations. Therefore, conformity and homogeneity comparisons were performed under matched target coverage conditions.

Statistical analysis was performed using the Wilcoxon signed-rank test to compare dosimetric parameters between ALDO and non-ALDO plans across different target separations. A *p*-value of less than 0.05 was considered statistically significant.

### 2.4. Patient Specific Quality Assurance and Gamma Analysis

Patient-specific QA was performed for the two-target configurations to evaluate delivery accuracy. Two verification methods were used: EPID-based PD and radiochromic film dosimetry.

**Figure 1:**
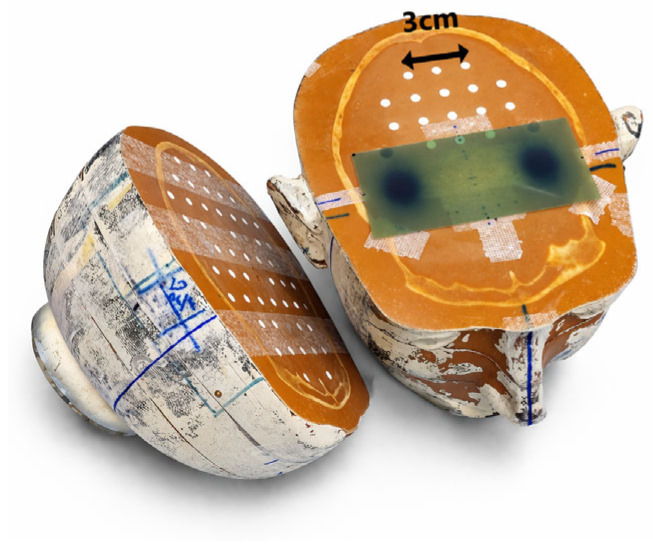
Anthropomorphic head phantom used for dosimetric verification. EBT4 radiochromic film was inserted between the phantom layers at the axial measurement plane. The center-to-center distance between the uniformly filled white circular inserts was fixed, as illustrated in the figure.

For PD, the measured fluence maps were compared with the calculated dose distributions from the treatment planning system. For film dosimetry, EBT4 radiochromic films were placed within the phantom to measure two-dimensional dose distributions, providing high spatial resolution for detecting dose variations, particularly in high-gradient regions (Figure 1). Each EBT4 film measurement was repeated at least three times for each plan to ensure measurement reproducibility and reduce experimental uncertainty. The film data were analyzed using DoseLab software (Version 7.0MR1, Varian Medical Systems, Palo Alto, CA, USA). Prior to measurement, the EBT4 films were calibrated using reference irradiations with known dose levels covering the clinical dose range used in this study. Films were scanned using an Epson Expression 10000XL flatbed scanner (Seiko Epson corp., Suwa City, Nagano, Japan) at 72 dpi resolution under consistent scanning conditions, including fixed orientation and color settings. To minimize variability associated with post-irradiation film response, all films were scanned 24 h after irradiation. The red color channel was used for dose analysis.

Due to the dose limitation of the EBT4 film (maximum measurable dose of approximately 10 Gy), patient-specific QA was performed only for plans generated without the ALDO function. This approach ensured that film measurements remained within the reliable dose response range [20,21]. Although some non-ALDO plans exhibited calculated maximum doses slightly above the nominal EBT4 limit, measured and calculated doses remained in acceptable agreement (1051.4 ± 10.31 cGy vs. 1040 cGy; deviation: 0.38–2.12%). In contrast, several ALDO configurations produced hotspot levels approaching 150% of the prescription dose, where film response uncertainty could not be reliably quantified.

Gamma analysis was performed to assess the agreement between measured and calculated dose distributions. Global dose normalization with a 10% dose threshold was applied for all gamma evaluations. Registration between measured and calculated dose distributions was initially performed using automatic image alignment in the software based on phantom geometry and film positioning markers, followed by manual fine adjustment to optimize spatial matching prior to gamma analysis. The evaluation criteria included 1 mm/3%, 1 mm/4%, 1 mm/5%, 1.5 mm/3%, and 2 mm/3%. The gamma passing rate was recorded for each measurement and used to evaluate the impact of tumor geometry and plan complexity on delivery accuracy. Comparisons were also made between PD and film results to assess differences in sensitivity between the two QA methods.

## 3. Results

### 3.1. Dosimetry Comparison

The results in Table 1 and Table 2 summarize the dosimetric parameters for target diameter of 1 cm and 2 cm, respectively. Overall, ALDO consistently improved dose conformity compared with non-ALDO across both target sizes and all inter-target distances. For the 1 cm targets, the RTOG conformity index was notably lower in ALDO plans (1.10–1.20) than in non-ALDO plans (1.15–1.40), with differences observed across all evaluated inter-target separation distances. A similar trend was observed for the 2 cm targets, where ALDO achieved lower RTOG CI values (1.01–1.15) compared with non-ALDO (1.18–1.21). The Paddick conformity index was also consistently higher and more stable in ALDO plans for both target sizes.

Regarding dose gradient, different behaviors were observed depending on target size. For the 1 cm targets, ALDO maintained lower and more stable GI values across increasing distances (3.33–4.27), whereas non-ALDO showed a substantial increase in GI at larger separations, particularly at 5–6 cm, indicating poorer dose fall-off. In contrast, for the 2 cm targets, GI values were comparable between the two techniques for most distances, with only minor differences observed, suggesting that the advantage of ALDO in dose gradient becomes less pronounced for larger targets.

In terms of dose homogeneity, ALDO plans exhibited higher ICRU83 HI values for both target sizes, indicating increased dose inhomogeneity. This effect was more prominent in the 1 cm targets, where HI values reached up to 0.40. Correspondingly, the maximum dose was consistently higher in ALDO plans, especially for the 1 cm targets at short distances, where values exceeded 150% of the prescription dose. For the 2 cm targets, the difference in maximum dose between ALDO and non-ALDO was reduced, with both techniques showing values around 120–130%.

### 3.2. Statistical Analysis

The statistical comparisons of dosimetric parameters between ALDO and non-ALDO plans for 1 cm and 2 cm targets are summarized in Table 3 and Table 4, respectively. Statistical analysis was performed using the Wilcoxon signed-rank test across different target separations.

For the 1 cm targets (Table 3), ALDO plans demonstrated significantly improved dosimetric conformity, including lower RTOG CI values, higher Paddick CI values, and lower GI values compared with non-ALDO plans (all *p* < 0.05). However, ALDO plans also exhibited significantly higher HI and maximum dose values (*p* < 0.05), indicating increased dose heterogeneity and hotspot formation in small, closely spaced targets.

For the 2 cm targets (Table 4), ALDO plans also achieved significantly improved conformity, with lower RTOG CI and higher Paddick CI values compared with non-ALDO plans (*p* < 0.05). In contrast to the 1 cm target group, no statistically significant differences were observed in GI or ICRU83 HI between the two techniques (GI: *p* = 0.916; HI: *p* = 0.844). Although ALDO plans showed lower maximum dose values than non-ALDO plans, the difference did not reach statistical significance (*p* = 0.063), suggesting that the hotspot effect associated with ALDO became less pronounced for larger targets.

### 3.3. Delivery Verification Results

**Figure 2:**
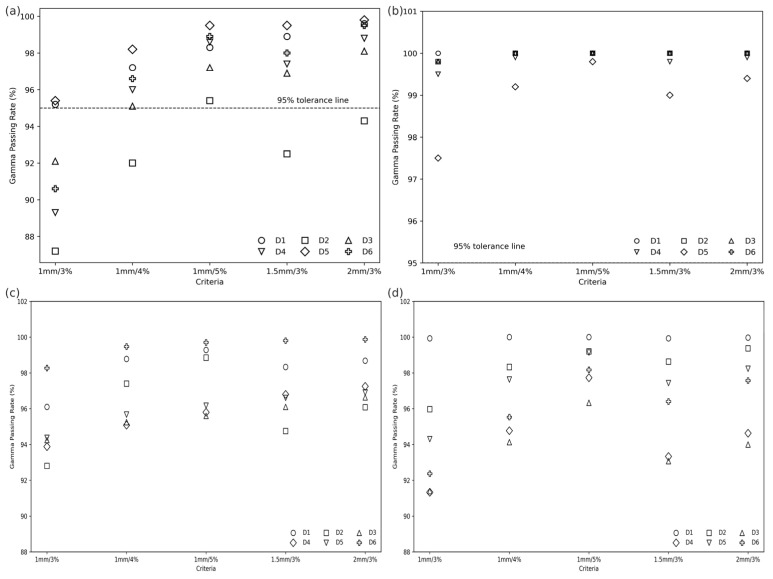
Spatial variation in gamma passing rates at different edge-to-edge inter-target separation (D1–D6) for targets with diameters of 1 cm and 2 cm. (**a**) Portal dosimetry (PD) results for 1 cm targets. (**b**) Portal dosimetry (PD) results for 2 cm targets. (**c**) Film measurements (mean values) for 1 cm targets. (**d**) Film measurements (mean values) for 2 cm targets. Gamma analysis was performed using criteria of 1 mm/3%, 1 mm/4%, 1 mm/5%, 1.5 mm/3%, and 2 mm/3%.

The comparison of gamma passing rates between portal dosimetry (PD) and film measurements for targets with diameters of 1 cm and 2 cm is presented in Table 5, Table 6 and Table 7. The variation in gamma passing rates across inter-target distance configurations (D1–D6, where D1 represents a 1 cm edge-to-edge separation between the two targets and D2–D6 represent progressively increasing separation distances) is further illustrated in Figure 2a,b.

For the 1 cm targets, the agreement between PD and film measurements varied across inter-target separation distance configurations and gamma criteria. For configurations with shorter inter-target separations (D1–D2), relatively good agreement was observed under less stringent gamma criteria, although noticeable discrepancies became evident under stricter criteria. In several cases, film measurements demonstrated higher gamma passing rates than PD, particularly at D2, where the PD–Film differences exceeded 5% under the 1 mm/3% and 1 mm/4% criteria (Table 7). Similar behavior was also observed at D6 under the 1 mm/3% criterion. These findings are reflected in Figure 2a, where reduced agreement between PD and film measurements was consistently observed under stricter gamma criteria.

For intermediate separation distance configurations (D3–D4), the differences between PD and film measurements became more variable, with both positive and negative deviations depending on the criteria. This mixed behavior suggests that the relationship between PD and film measurements was influenced by both inter-target separation configuration and evaluation criteria. In contrast, for configurations with larger separation distances (D5–D6), larger discrepancies were identified. The most pronounced difference was observed at D6 under the 1 mm/3% criterion, where the PD–Film difference exceeded 7%, indicating substantial disagreement between PD and film measurements in high-gradient regions (Table 7). As shown in Figure 2a, the discrepancies between PD and film measurements became more evident under stricter gamma criteria, particularly in configurations associated with greater off-axis displacement.

For the 2 cm targets, overall gamma passing rates were generally higher, particularly for configurations associated with shorter inter-target separations and smaller off-axis displacement, where both PD and film measurements approached 100%, indicating a ceiling effect (Table 6). However, for configurations associated with larger separation distances and greater off-axis displacement, the discrepancy between PD and film measurements increased.

For the D3 and D4 inter-target separation configurations, PD consistently yielded higher gamma passing rates than film measurements, with differences ranging from approximately 5% to 8% across multiple criteria. A similar trend was observed for the D6 configuration, where the differences remained pronounced, particularly under stricter criteria (Table 7). These findings indicate systematic differences between PD and film measurements in configurations associated with greater off-axis displacement, which may reflect the distinct detector characteristics and spatial resolution of the two QA methodologies. These findings are clearly illustrated in Figure 2b, which demonstrates a systematic reduction in film-based gamma passing rates relative to PD under greater off-axis conditions.

Overall, Table 5, Table 6 and Table 7 and Figure 2 demonstrate a clear spatial dependence of the agreement between PD and film measurements on inter-target separation configuration and target geometry. Larger discrepancies were observed for configurations with larger inter-target separations (D3–D6) compared with configurations involving shorter separations (D1–D2). This effect was more pronounced for the 2 cm targets, suggesting that both target size and geometric configuration influence the reliability of PD-based verification. The corresponding film-based distributions are presented in Figure 2c,d, showing the mean gamma passing rates across different inter-target separation distance configurations.

## 4. Discussion

The present study demonstrates that tumor geometric configuration plays a critical role in determining both dosimetric performance and delivery accuracy in HyperArc-based multi-target SRS. In particular, inter-target separation distance, target size, and off-axis target location were found to significantly influence dose conformity, gradient characteristics, and verification outcomes.

From a planning perspective, the use of the ALDO function consistently improved conformity across all geometric configurations. This finding agrees with previous studies reporting that HyperArc-based automated optimization enhances conformity and reduces inter-planner variability [5,6,7,8,9]. The relatively stable RTOG and Paddick conformity indices observed in ALDO plans suggest that automated lower dose objectives provide a robust and efficient optimization framework for complex multi-target scenarios.

However, the improved conformity achieved with ALDO was accompanied by an increase in maximum dose within the target, particularly for small targets with short inter-target distances. In SRS treatment planning, a certain degree of dose heterogeneity is generally expected because of the ablative treatment intent. Therefore, the absolute magnitude of the maximum dose may be more clinically relevant than heterogeneity itself. In the present study, maximum doses exceeding 150% of the prescription were observed in closely spaced targets, indicating a substantial concentration of dose within limited regions.

From a clinical perspective, elevated dose concentrations may reflect increased local dose accumulation within closely spaced target configurations. Korytko et al. demonstrated that the volume of normal brain receiving 12 Gy (V12) is a significant predictor of radionecrosis following Gamma Knife radiosurgery [22], and similar dose–volume relationships have been reported in subsequent studies. Although V12 was not directly evaluated in the present study, closely spaced lesions demonstrated increased overlap of intermediate- and high-dose regions within the phantom configurations evaluated. Furthermore, the elevated maximum doses observed in this study may reflect increased local dose concentration within closely spaced target configurations, potentially increasing dose spill into adjacent regions surrounding the targets. This phenomenon may be associated with dose bridging between adjacent targets, where individual high-dose regions merge and reduce spatial dose separation. Therefore, these observations should be interpreted as dosimetric findings derived from simplified phantom configurations, suggesting that careful evaluation of maximum dose and normal brain dose-volume characteristics may be important when applying ALDO in cases with limited inter-target separation, rather than as direct predictors of clinical radionecrosis risk.

The influence of tumor geometry was further reflected in dose gradient behavior. For small targets, increasing inter-target distance was associated with improved gradient characteristics, whereas closely spaced targets exhibited less favorable dose fall-off. This finding may be related to overlapping dose distributions between adjacent lesions, which can increase low-dose spread and reduce gradient sharpness, particularly in closely spaced targets [13,14]. From a physical perspective, this phenomenon may partially relate to overlapping beam apertures and reduced modulation flexibility when multiple targets share a single isocenter, potentially limiting the optimizer’s ability to independently shape steep dose gradients.

In terms of delivery verification, a systematic difference between portal dosimetry and film measurements was observed. For the 2 cm targets, portal dosimetry generally demonstrated higher gamma passing rates than film measurements, particularly under stricter criteria and in configurations involving greater target off-axis displacement from the isocenter. However, the 1 cm targets showed mixed behavior depending on target configuration, off-axis geometry, and gamma criteria. This finding is consistent with previous studies reporting limitations of EPID-based QA in accurately capturing complex dose distributions [15]. In contrast, radiochromic films demonstrate greater sensitivity to spatial dose variations, particularly in high-gradient regions. EPID portal dosimetry and film dosimetry are not fully equivalent QA methodologies. EPID-based verification primarily evaluates integrated detector response and fluence agreement, whereas film dosimetry provides high-spatial-resolution absorbed dose measurements. Therefore, the observed differences between PD and film measurements in this study likely reflect intrinsic differences in detector sensitivity and spatial sampling characteristics rather than direct superiority of one QA system over the other.

Notably, larger discrepancies between PD and film measurements were observed in configurations with greater off-axis target displacement and under stricter gamma criteria, suggesting spatial dependence of delivery accuracy. This observation is consistent with previous reports showing that both targeting and dosimetric uncertainties increase at off-axis locations, even with modern image-guided systems [11,12]. Furthermore, the fixed gantry and couch angle configuration inherent to HyperArc may impose additional limitations on dose delivery and verification; in particular, when the spatial distribution of targets is aligned parallel to the gantry rotation axis, the available beam modulation and angular sampling may be constrained, potentially leading to reduced QA sensitivity in certain regions. These findings further support the notion that tumor geometric distribution plays a critical role in influencing QA outcomes, suggesting that certain geometric arrangements are more susceptible to measurement discrepancies and highlighting that reliance on central-axis or single-point QA alone may be insufficient for comprehensive evaluation of complex multi-target SRS plans, warranting further investigation in future studies.

From a clinical standpoint, these findings emphasize that tumor geometry should be considered an important factor in treatment planning and QA strategy selection. In cases involving closely spaced targets, additional attention should be given to maximum dose control, potential dose bridging, and normal brain dose exposure. The integration of multi-positional and high-resolution QA approaches, such as film-based verification, may provide a more comprehensive assessment of delivery accuracy in these scenarios.

Several limitations should be acknowledged. First, this study was conducted using an anthropomorphic phantom and therefore does not fully account for patient-specific anatomical heterogeneity. Second, film-based QA was limited to non-ALDO plans because of film dose–response limitations, which may restrict direct comparison between optimization strategies. Therefore, the QA findings should be interpreted as reflecting non-ALDO delivery verification only. Third, only two-target configurations were evaluated, and more complex multi-target geometries may exhibit additional dosimetric interactions.

Fourth, the statistical analysis was performed using only six paired geometric configurations (*n* = 6). Consequently, the Wilcoxon signed-rank test had limited statistical resolution, and statistically significant findings primarily reflect consistent directional differences across configurations rather than strongly powered inferential evidence.

Fifth, because this study employed a homogeneous phantom design, the influence of tissue heterogeneity on AAA dose calculation accuracy was expected to be limited. Nevertheless, the use of AAA instead of AcurosXB or Monte Carlo remains a limitation of this study, particularly for small-field high-gradient SRS dose calculations. Future studies incorporating patient datasets, advanced dose calculation algorithms, more complex multi-target arrangements, and three-dimensional dosimetry are warranted to further validate these findings.

## 5. Conclusions

Within the simplified two-target phantom configurations evaluated in this study, tumor geometric distribution plays a significant role in both dosimetric performance and delivery verification in HyperArc-based multi-target stereotactic radiosurgery. Increasing inter-target distance was associated with improved dose conformity and gradient characteristics, whereas closely spaced targets resulted in greater intermediate dose spread and potential dose bridging. The application of the ALDO function improved conformity consistency across different geometric configurations; however, this benefit was accompanied by elevated maximum dose levels, particularly in cases with limited inter-target separation, suggesting that careful dosimetric evaluation may be warranted when applying ALDO in small or closely spaced target configurations.

In delivery verification, the agreement between portal dosimetry and film measurements varied depending on target size, gamma criteria, and spatial position. In the non-ALDO configurations evaluated in this study, portal dosimetry generally demonstrated higher gamma passing rates for the 2 cm targets, whereas mixed results were observed for the 1 cm targets. In contrast, film-based dosimetry demonstrated greater sensitivity to spatial dose variations. Therefore, supplemental film-based QA may be beneficial for complex geometric configurations where accurate spatial dose verification is clinically important.

Overall, these findings highlight the importance of incorporating tumor geometry into treatment planning and QA strategy selection for simplified multi-target SRS configurations. These observations may be particularly relevant in cases involving closely spaced targets and high-dose gradient regions. In addition, the integration of multiple QA tools or the use of systems capable of multi-positional verification should be considered to improve the completeness and accuracy of dose delivery assessment.

## Figures and Tables

**Table 1:** Comparison of dosimetric parameters for different inter-target distances using ALDO and non-ALDO planning (target diameter = 1 cm).

Distance (cm)	Technique	RTOG CI	Paddick CI	GI	ICRU83 HI	Max Dose (%)
1	ALDO	1.20	0.79	3.44	0.40	158.1
2	ALDO	1.20	0.79	3.33	0.40	157.4
3	ALDO	1.19	0.79	3.51	0.34	146.5
4	ALDO	1.16	0.8	3.95	0.24	129.0
5	ALDO	1.14	0.81	4.02	0.18	121.1
6	ALDO	1.10	0.81	4.27	0.17	118.6
1	Non-ALDO	1.37	0.71	3.55	0.19	130.3
2	Non-ALDO	1.39	0.71	3.36	0.18	130.2
3	Non-ALDO	1.40	0.71	3.59	0.19	129.8
4	Non-ALDO	1.30	0.75	4.81	0.10	114
5	Non-ALDO	1.15	0.8	6.14	0.08	108.8
6	Non-ALDO	1.19	0.78	5.64	0.08	109.8

**Table 2:** Comparison of dosimetric parameters for different inter-target distances using ALDO and non-ALDO planning (target diameter = 2 cm).

Distance (cm)	Technique	RTOG CI	Paddick CI	GI	ICRU83 HI	Max Dose (%)
1	ALDO	1.11	0.88	6.25	0.19	126.0
2	ALDO	1.02	0.89	3.03	0.23	126.1
3	ALDO	1.12	0.87	2.84	0.17	124.2
4	ALDO	1.15	0.86	2.84	0.19	127.4
5	ALDO	1.11	0.88	3.02	0.13	118.4
6	ALDO	1.10	0.88	3.03	0.14	117.8
1	Non-ALDO	1.18	0.84	6.44	0.16	125.4
2	Non-ALDO	1.21	0.83	2.89	0.21	130.4
3	Non-ALDO	1.21	0.83	2.93	0.19	130.3
4	Non-ALDO	1.20	0.83	2.94	0.20	130.2
5	Non-ALDO	1.21	0.83	2.92	0.17	126.9
6	Non-ALDO	1.20	0.83	3.00	0.15	122.7

**Table 3:** Statistical comparison of dosimetric parameters between ALDO and non-ALDO plans for 1 cm diameter targets.

Dosimetric Parameter	ALDO (Mean ± SD)	Non-ALDO (Mean ± SD)	*p*-Value
RTOG CI	1.17 ± 0.04	1.30 ± 0.11	**0.031**
Paddick CI	0.80 ± 0.01	0.75 ± 0.04	**0.03** **4**
GI	3.75 ± 0.38	4.52 ± 1.19	**0.031**
ICRU83 HI	0.29 ± 0.10	0.14 ± 0.05	**0.031**
Max Dose (%)	138.5 ± 17.9	120.5 ± 10.6	**0.031**

*p*-values were obtained from the Wilcoxon signed-rank test based on six paired geometric configurations (*n* = 6). Bold values indicate statistical significance (*p* < 0.05).

**Table 4:** Statistical comparison of dosimetric parameters between ALDO and non-ALDO plans for 2 cm diameter targets.

Dosimetric Parameter	ALDO (Mean ± SD)	Non-ALDO (Mean ± SD)	*p*-Value
RTOG CI	1.10 ± 0.04	1.20 ± 0.01	**0.03** **6**
Paddick CI	0.88 ± 0.01	0.83 ± 0.00	**0.03** **5**
GI	3.50 ± 1.36	3.52 ± 1.44	0.916
ICRU83 HI	0.18 ± 0.04	0.18 ± 0.02	0.844
Max Dose (%)	123.3 ± 4.3	127.7 ± 3.2	0.063

*p*-values were obtained from the Wilcoxon signed-rank test based on six paired geometric configurations (*n* = 6). Bold values indicate statistical significance (*p* < 0.05).

**Table 5:** Comparison of gamma passing rates (%) between portal dosimetry (PD) and film measurements for non-ALDO plans at different edge-to-edge inter-target separation (target diameter = 1 cm).

Distance (cm)	Verification Tool	1 mm/3%	1 mm/4%	1 mm/5%	1.5 mm/3%	2 mm/3%
1	EPID-PD	95.2	97.2	98.3	98.9	99.6
2	EPID-PD	87.2	92.0	95.4	92.5	94.3
3	EPID-PD	92.1	95.1	97.2	96.9	98.1
4	EPID-PD	89.3	96.0	98.6	97.4	98.8
5	EPID-PD	95.4	98.2	99.5	99.5	99.8
6	EPID-PD	90.6	96.6	98.9	98.0	99.5
1	Film	96.10 ± 1.48	98.78 ± 1.66	99.28 ± 1.45	98.33 ± 1.22	98.68 ± 1.13
2	Film	92.80 ± 4.89	97.40 ± 1.62	98.85 ± 1.20	94.75 ± 3.28	96.08 ± 1.81
3	Film	94.25 ± 6.44	95.25 ± 6.40	95.60 ± 6.08	96.10 ± 4.88	96.63 ± 4.46
4	Film	93.88 ± 6.36	95.08 ± 5.97	95.80 ± 5.87	96.80 ± 5.28	97.25 ± 4.98
5	Film	94.37 ± 5.02	95.67 ± 5.20	96.17 ± 5.16	96.60 ± 4.81	96.90 ± 4.45
6	Film	98.27 ± 2.10	99.47 ± 0.92	99.70 ± 0.52	99.80 ± 0.26	99.87 ± 0.15

**Table 6:** Comparison of gamma passing rates (%) between portal dosimetry (PD) and film measurements for non-ALDO plans at different edge-to edge inter-target separation (target diameter = 2 cm).

Distance (cm)	Verification Tool	1 mm/3%	1 mm/4%	1 mm/5%	1.5 mm/3%	2 mm/3%
1	EPID-PD	100.0	100.0	100.0	100.0	100.0
2	EPID-PD	99.8	100.0	100.0	100.0	100.0
3	EPID-PD	99.8	100.0	100.0	100.0	100.0
4	EPID-PD	99.5	99.9	100.0	99.8	99.9
5	EPID-PD	97.5	99.2	99.8	99.0	99.4
6	EPID-PD	99.8	100.0	100.0	100.0	100.0
1	Film	99.93 ± 0.12	100.00 ± 0.00	100.00 ± 0.00	99.93 ± 0.12	99.97 ± 0.06
2	Film	95.97 ± 1.85	98.33 ± 0.85	99.20 ± 0.46	98.63 ± 0.55	99.37 ± 0.45
3	Film	91.40 ± 5.28	94.13 ± 4.39	96.33 ± 3.00	93.07 ± 4.62	94.00 ± 4.07
4	Film	91.33 ± 2.74	94.77 ± 2.63	97.73 ± 1.89	93.33 ± 2.57	94.63 ± 2.48
5	Film	94.30 ± 0.35	97.63 ± 0.46	99.13 ± 0.81	97.43 ± 1.10	98.23 ± 0.58
6	Film	92.37 ± 5.69	95.53 ± 4.68	98.17 ± 2.22	96.40 ± 2.72	97.57 ± 1.88

**Table 7:** The difference between PD and the mean film value (Difference = PD − Film mean).

Distance (cm)	Tumor Diameter (cm)	Diff. 1 mm/3%	Diff. 1 mm/4%	Diff. 1 mm/5%	Diff. 1.5 mm/3%	2 mm/3%
1	1	−0.9	−1.58	−0.98	0.57	0.92
2	1	−5.60	−5.40	−3.43	−2.25	−1.78
3	1	−2.15	−0.15	1.60	0.80	1.47
4	1	−4.58	0.92	2.80	0.60	1.55
5	1	1.03	2.53	3.33	2.90	2.90
6	1	−7.67	−2.87	−0.80	−1.80	−0.37
1	2	0.07	0.00	0.00	0.57	0.03
2	2	3.83	1.67	0.80	1.37	0.63
3	2	8.40	5.87	3.67	6.93	6.00
4	2	8.17	5.13	2.27	6.47	5.27
5	2	3.20	1.57	0.67	1.57	1.17
6	2	7.43	4.47	1.83	3.60	2.43

## Data Availability

The data that support the findings of this study are available from the corresponding author upon reasonable request. Due to patient privacy and institutional policy restrictions, the data are not publicly available.

## Abbreviations

The following abbreviations are used in this manuscript:

ALDO	Automatic Lower Dose Objective
QA	Quality Assurance
EPID	Electronic Portal Imaging Device
PD	Portal Dosimetry
CT	Computed Tomography
SRS	Stereotactic Radiosurgery
SIMT	Single Isocenter Multi Target
VMAT	Volumetric Modulated Arc Therapy
AAA	Anisotropic Analytical Algorithm
CI	Conformity Index
GI	Gradient Index
HI	Homogeneity Index
